# Empowering the elderly mind: Mindfulness as a tool to improve psychological resilience and quality of life

**DOI:** 10.6026/973206300212661

**Published:** 2025-08-31

**Authors:** Gomathi Sivaprakash, Venkatesh Mathankumar Vasudevan, Shankar Shanmugam Rajendran, Marudan Anbalagan, Jayalakshmi Lakhmanan, Elilarasi Mani, Packiyalakshmi Muthuchy

**Affiliations:** 1 Department of Mental Health Nursing, College of Nursing, Madras Medical College, The Tamil Nadu Dr.MGR Medical University, Chennai, Tamil Nadu, India; 2 Department of Mental Health, Institute of Mental Health, Kilpauk, The Tamil Nadu Dr.MGR Medical University, Chennai, Tamil Nadu, India; 3 Department of Child Health Nursing, College of Nursing, Madras Medical College, The Tamil Nadu Dr.MGR Medical University, Chennai, Tamil Nadu, India

**Keywords:** Quality of life, psychological well-being, mindfulness based stress reduction, geriatrics

## Abstract

The effectiveness of Mindfulness-Based Stress Reduction (MBSR) in enhancing the quality of life and mental health of older adults is of interest. A quasi-experimental design with 60 participants was used, with an experimental group undergoing MBSR and a control group receiving routine care. Results showed a significant positive correlation between improved quality of life and psychological well-being in the experimental group (r = 0.46, p = 0.01). The control group exhibited poor correlation (r = 0.15, p = 0.32). MBSR significantly improved both domains, demonstrating its effectiveness in enhancing elderly mental health and quality of life.

## Background:

Aging naturally involves gradual declines in cellular regeneration, organ function and physical, psychological and social capacities, often leading to increased health issues, emotional distress and loneliness among older adults [1]. With global life expectancy on the rise, the goal has shifted from simply extending lifespan to enriching the quality of the added years [2]. Mindfulness Based Stress Reduction (MBSR), incorporating meditation, body scans, mindful movement and non-judgmental present moment awareness, has been shown to be feasible and well accepted in elderly populations [3]. Research indicates it alleviates symptoms of stress, anxiety, depression, sleep disturbances and chronic pain in older adults, while also fostering emotional regulation, resilience and cognitive functions like memory and attention [4]. These psychosocial and bio psychological benefits contribute to improved well-being and life satisfaction, positioning MBSR as a holistic, accessible strategy to support dignified and fulfilling aging [5]. Therefore, it is of interest to explore the effectiveness of Mindfulness-Based Stress Reduction (MBSR) in enhancing the quality of life and mental health of older adults.

## Methodology:

This study utilized a quantitative Quasi experimental design to evaluate the impact of Mindfulness Based Stress Reduction on Quality of Life and Psychological well-being among elderly attended in National centre of Aging, Guindy and Chennai. Approved by the Institutional Ethics Committee, (IEC-MMC/Approval/36112024). 60 participants were included in the study by using Non-Probability convenience sampling technique. The participants are equally divided into an experimental group and control group, among that the experimental group received Mindfulness based stress reduction for 4 weeks such as body scan, mindfulness eating, mindfulness walking, mindfulness listening of song and a control group continued with routine care. Data were collected using the OPQOLBRIEF Questionnaire (13 Items) and RYFF psychological well-being scale (18Items). Pre and post intervention analysed using SPSS version. Outcomes compared with chi-square and independent t test, aimed to measure the benefits of the intervention on quality of life and psychological wellbeing.

## Results:

The majority of participants in both the experimental and control groups were equally male and female, with 50% each. Most participants were between 60-70 years old, with 63.34% in the experimental group and 53.33% in the control group. High school education was the most common level attained in both groups. Hinduism was the predominant religion (80%), and most participants were married 96.67% in the experimental group and 93.33% in the control group. The main source of income was support from children, and most lived in nuclear families. Diabetes was the most common health issue in the control group, while both diabetes and hypertension were equally common in the experimental group. In terms of quality of life, the experimental group showed a substantial improvement, with the mean score increasing from 38.20 (58.77%) in the pretest to 53.20 (81.85%) reflecting a gain of 23.08%. In contrast, the control group exhibited only a minor increase from 39.53 (6.85%) in the posttest 0.82%) to 41.40 (63.69%), resulting in a gain of 2.87%. The experimental group demonstrated notable progress, with the mean score increasing from 60.00 (47.62%) to 95.20 (75.56%), representing a gain of 35.20%. The control group showed a marginal improvement from 61.03 (48.44%) to 63.43 (50.34%), indicating an increase of only 1.90%. There is a significant positive moderate correlation between posttest QOL score and posttest psychological well-being score. It means QOL score increases their perceived psychological well-being score also increases moderately. Association between posttest level of QOL score and Psychological well-being score with demographic variables indicated that age, education, and pensioner were significant predictor for improving quality of life and psychological well-being. The correlation between posttest QOL scores and psychological well-being scores in both the experimental and control groups was analyzed. In the experimental group, a significant moderate positive correlation was found (r = 0.46, p = 0.01), indicating that as the QOL score increased (mean = 53.20 ± 3.81), the psychological well-being score also increased (mean = 95.20 ± 3.77) (Table 1 - see PDF). On the other hand, in the control group, the correlation was weak and not statistically significant (r = 0.15, p = 0.32), suggesting a poor positive relationship between the QOL score (mean = 41.40 ± 3.41) and psychological well-being score (mean = 63.43 ± 3.18) (Table 2 - see PDF).

## Discussion:

The current study evaluated the effectiveness of Mindfulness-Based Stress Reduction (MBSR) on quality of life and psychological well-being among participants in experimental and control groups. In terms of quality of life, the experimental group showed a substantial improvement, with the mean score increasing from 38.20 (58.77%) in the pretest to 53.20 (81, reflecting a gain of 23.08%. In contrast, the control group exhibited only a minor increase from 39.53 (6.85%) in the posttest 0.82%) to 41.40 (63.69%), resulting in a gain of 2.87%. Similarly, in psychological well-being, the experimental group demonstrated notable progress, with the mean score rising from 60.00 (47.62%) to 95.20 (75.56%), accounting for a gain of 27.94%. The control group showed a marginal improvement from 61.03 (48.44%) to 63.43 (50.34%), indicating a gain of only 1.90%. These findings highlighted the significant positive impact of MBSR on enhancing both quality of life and psychological well-being. These study findings were supported by a similar study conducted by Lakshmanan *et al.* (2024) [6] on mind-body interventions and psychosocial and bio-physiological markers among the elderly. The results showed improvements in Quality of Life and reductions in stress and depression among the elderly in a selected old-age home in Chennai. Moreover, the findings from this study also align with other research that has shown how MBSR can enhance cognitive functions, such as attention and memory, and improve emotional resilience in older adults. For example, studies by Reibel *et al.* (2001) [7] and Keng (2011) *et al.* [8] have similarly reported improvements in psychological well-being and reduced symptoms of anxiety, depression, and stress following MBSR interventions in older populations. These studies underscore the growing recognition of MBSR as an effective, holistic approach to addressing the mental health challenges of aging. The findings from the experimental and control groups in the current study also suggest that the positive effects of MBSR are not just statistically significant but also practically meaningful, as indicated by the substantial improvements in the experimental group compared to the minor increases observed in the control group. This highlights the value of structured, mindfulness-based interventions in producing tangible, measurable benefits for the elderly, thereby supporting the broader push for integrative health strategies aimed at improving aging populations' quality of life.

## Conclusion:

Mindfulness based stress reduction is a highly effective intervention for improving quality of life and psychological well-being among elderly patients. The participants who underwent structured Mindfulness based stress reduction session showed significant changes in quality of life and psychological well-being. Compare to those who received on routine care.
